# Biochemical Mechanisms and Rehabilitation Strategies in Osteoporosis-Related Pain: A Systematic Review

**DOI:** 10.3390/clinpract14060216

**Published:** 2024-12-19

**Authors:** Giorgia Natalia Iaconisi, Rachele Mancini, Vincenzo Ricci, Danilo Donati, Cristiano Sconza, Riccardo Marvulli, Maurizio Ranieri, Marisa Megna, Giustino Varrassi, Simone Della Tommasa, Andrea Bernetti, Loredana Capobianco, Giacomo Farì

**Affiliations:** 1 Department of Biological and Environmental Sciences and Technologies, University of Salento (DiSTeBA), 73100 Lecce, Italy; giorgianatalia.iaconisi@unisalento.it (G.N.I.); loredana.capobianco@unisalento.it (L.C.); 2 Department of Translational Biomedicine and Neuroscience (DiBraiN), Aldo Moro University, 70125 Bari, Italy; mancinirachelerosaria@gmail.com (R.M.); riccardo.marvulli@policlinico.ba.it (R.M.); maurizio.ranieri@uniba.it (M.R.); marisa.megna@uniba.it (M.M.); 3 Physical and Rehabilitation Medicine Unit, Luigi Sacco University Hospital, ASST Fatebenefratelli-Sacco, 20157 Milan, Italy; ricci.vincenzo@asst-fbf-sacco.it; 4 Clinical and Experimental Medicine PhD Program, University of Modena and Reggio Emilia, 41125 Modena, Italy; danilo.donati@unimore.it; 5 IRCCS Humanitas Research Hospital, 20089 Milan, Italy; cristiano.sconza@humanitas.it; 6 Fondazione Paolo Procacci, 00193 Roma, Italy; giuvarr@gmail.com; 7 Department for Horses, University of Leipzig, 04103 Leipzig, Germany; della.tommasa@vetmed.uni-leipzig.de; 8 Department of Experimental Medicine (Di.Me.S), University of Salento, 73100 Lecce, Italy

**Keywords:** osteoporosis, pain, pathway alteration, metabolic pathways, rehabilitation

## Abstract

Background/Objectives: Osteoporosis causes a bone mass reduction and often determines acute and chronic pain. Understanding the biochemical and neurophysiological mechanisms behind this pain is crucial for developing new, effective rehabilitative and therapeutic approaches. This systematic review synthesizes recent advances in muscle–bone interactions and molecular pathways related to osteoporosis-associated pain. Methods: We carried out a systematic review including studies published from 2018 to 2024 using PubMed, Scopus, clinicaltrials.gov and Cochrane Library. The Cochrane Collaboration tool was used to assess bias risk. The review adhered to PRISMA guidelines and is registered with PROSPERO (CRD42024574456); Results: Thirteen studies were included. It emerged that osteoporosis causes progressive bone loss due to disruptions in biochemical processes and muscle–bone interactions. This condition is also closely associated with the development of pain, both acute and chronic. Key findings include the role of the miR-92a-3p/PTEN/AKT pathway and the impact of muscle–bone disconnection on bone health. Mechanotransduction is critical for bone maintenance. Effective pain management and rehabilitation strategies include physical therapy and physical exercise, yoga, Pilates, and cognitive behavioral therapy (CBT); they all improve pain relief and functional outcomes by enhancing muscle strength, flexibility, and balance. Pharmacological options such as NSAIDs, opioids, and new agents like SHR-1222, along with surgical interventions like percutaneous vertebroplasty, offer additional pain reduction, especially when included in individualized rehabilitation projects; Conclusions: This review highlights advancements in understanding osteoporotic pain mechanisms and identifies promising treatments. Integrating targeted therapies and rehabilitation strategies can enhance patients’ pain relief.

## 1. Introduction

Osteoporosis (OP) is a widespread metabolic systemic bone disease characterized by increased bone fragility, low bone mass, and a heightened risk of fractures, and it significantly impacts individuals’ health and quality of life [1]. This condition poses a substantial public health challenge, accounting for over 8.9 million fractures annually worldwide, translating to 1 fracture every three seconds and affecting more than 200 million people globally [1]. OP often remains asymptomatic until a fracture occurs, typically involving vulnerable areas like the wrist, hip, or spine; such injuries may even occur as a result of mundane activities such as sneezing or coughing [1]. Vertebral fractures can lead to acute and chronic pain, further exacerbating the burden of OP on daily activities. Although age-related bone loss is a primary concern, OP affects individuals across all ages and genders, with women, especially post-menopause, facing heightened susceptibility [2]. Beyond age and hormonal factors, numerous variables, including drugs, medical conditions and lifestyle choices, contribute to the development and progression of OP [2]. Prompt detection through bone density scans and timely initiation of bone-strengthening therapies are critical steps for managing the disease and averting debilitating fractures.

While OP is often asymptomatic until a fracture occurs, pain is a common clinical manifestation and a significant burden for affected individuals. OP-related pain can arise from multiple sources, including acute fractures, particularly vertebral fractures, which are prevalent and can lead to sudden and severe back pain [3]. Furthermore, in the scientific community, there is an increasingly widespread hypothesis that chronic pain could develop due to persistent changes in bone structure, joint involvement and muscle strain resulting from altered biomechanics and decreased bone density [4]. In fact, pain also seems to be a result of OP-induced changes in bone micro-architecture contributing to a chronic evolution. Specifically, reduced bone density and compromised bone quality increase the amount of mechanical stress on bones, leading to microfractures and microdamage. These structural alterations chronically stimulate pain receptors and inflammatory mediators, perpetuating pain perception [5]. Moreover, OP can affect the joints adjacent to the weakened bones, such as hips and knees. Degenerative changes in these weight-bearing joints contribute to pain and stiffness, further complicating mobility and functional impairment in the affected individuals [6].

This means that weakened bones and altered posture due to OP force muscles to work harder to maintain balance. This increased muscular effort can lead to chronic muscle strain, particularly in the back and lower extremities, exacerbating pain symptoms [7].

Moreover, it is well known that structural changes associated with vertebral fractures or bone deformities can compress the adjacent nerves, causing radicular pain and neuropathic symptoms. Nerve compression further amplifies pain perception and may lead to sensory disturbances in the affected areas [8].

Understanding the genetic predispositions and molecular pathways involved in bone metabolism provides valuable insights into individual susceptibility to OP and could help to improve our understanding of the mechanisms underlying OP-related chronic pain, with the final aim of preventing it. In this sense, the identification of specific genetic markers and signaling pathways associated with bone homeostasis and its alterations due to OP could offer new opportunities for targeted therapeutic interventions. 

Despite significant advancements in our understanding of the pathophysiology of OP and associated pain, several gaps remain in the literature. As highlighted by Mattia et al., 2016, numerous studies have explored the mechanisms behind bone pain in osteoporosis [9]. It has been shown that osteoporotic pain is influenced by both peripheral mechanisms, such as the sensitization of sensory nerve fibers in the bones, and central mechanisms, which include the activation of receptors in the central nervous system that amplify pain [9,10]. Specific receptors, such as TRPV1 and ASIC-3, have been identified as key players in this process, along with the relationship between sympathetic and sensory nerves [11,12]. This has led to the suggestion that drugs like beta blockers could modulate pain. Furthermore, central sensitization plays a major role in chronic pain, involving microglia and neuropeptides. Managing osteoporotic pain remains complex and may require multimodal approaches, including opioids, although careful attention must be paid to side effects, such as androgen deficiency [9]. 

This review focuses on the biochemical basis of OP, emphasizing the underlying mechanisms of OP-related pain and the rehabilitation strategies that can relieve this condition. By exploring crucial signaling pathways involved in bone metabolism and pain perception, this review aims to highlight how dysregulation could contribute significantly to increased bone resorption by osteoclasts and decreased bone formation by osteoblasts, leading to heightened pain experiences. Additionally, the role of inflammatory markers, genetic factors and altered biomechanics in chronic pain associated with OP is examined. Finally, this review discusses emerging rehabilitation strategies, including advanced physical therapy techniques, pharmacological interventions, and novel therapeutic targets, aimed at managing OP-related pain and improving patients’ quality of life. This comprehensive approach sets the stage for future research and personalized treatments to mitigate the debilitating impact of OP.

## 2. Materials and Methods

### 2.1. Search Strategy

This systematic review was carried out according to the Preferred Reporting Items for Systematic Reviews and Meta-Analyses (PRISMA) statement guidelines (Prospero registration-2024, n: CRD42024574456). The objective of our review was to describe the current evidence in the literature about the pathophysiology of pain in OP, describing its possible molecular pathways and how they affect the management of this important pathology in terms of therapies and rehabilitation strategies.

Several research questions that would meet the objective of the review were identified: What are the molecular pathways involved in OP-related pain? In which way do they affect bone metabolism? How can they be usefully modulated in the treatment and rehabilitation of this disease?

To address these questions, a systematic search was performed between April 2024 and August 2024 using PubMed, Cochrane Library, ClinicalTrials.gov and Web of Science (WoS). The same search equation was applied consistently across all databases to ensure uniformity and comprehensive retrieval of relevant studies. The Boolean syntax used for all the databases was (“Osteoporosis” AND (“pain” OR “pain pathways” OR “osteoporotic pain” OR “osteoporosis treatment”)). The search was limited to publications from the past six years (January 2018–August 2024), ensuring that only the most recent and innovative evidence would be included, reflecting advances in the understanding and management of OP-related pain and the related therapeutic interventions. 

The following filters were applied across all databases:

- Text availability: full text;
- Species: humans;
- Language: English.

By using a consistent search equation across multiple platforms, this study aimed to systematically identify high-quality, up-to-date research addressing the abovementioned questions. The methodology ensures transparency and reproducibility of the search process, providing a robust foundation for the synthesis of evidence.

### 2.2. Data Collection Process and Identification of the Selection Criteria

Three independent authors conducted the search and compared their findings. Any disagreements were resolved by consensus, involving three additional experienced authors. After collecting potentially eligible articles, further selection was performed based on the inclusion and exclusion criteria. 

The inclusion criteria were as follows:

- Studies published in English that involved patients with OP and focused on the resulting pain and its biochemical etiology;
- Full-text availability;
- No age restriction.

The exclusion criteria were as follows:

- Studies that did not address the research questions outlined in this review;
- Articles that did not meet the basic requirements for experimental study design, such as case reports, review studies, meta-analyses, case studies, practical guidelines, books and unpublished or retracted studies.

Although the inclusion and the exclusion criteria were integrated with the search strategy filters to streamline the selection process, we explicitly stated these criteria in the methodology section to enhance the transparency, clarity and reproducibility of the review process.

### 2.3. Analysis of Methodological Quality and Risk of Bias

The methodological quality of the included studies was evaluated using a modified version of the Jadad Quality Scale. The scale was used to assess various aspects of the studies, such as randomization, the description of withdrawals, and the statistical methods employed.

In line with Cochrane recommendations, the Risk-of-Bias 2 (RoB 2) tool was applied to systematically evaluate potential biases across several domains. This tool assesses biases in areas including randomization, adherence to interventions, handling of missing data, outcome measurement, and the selection of reported results. The evaluation was performed according to the guidelines of Sterne et al. (2019) [13], ensuring structured and transparent assessment of methodological quality.

The RoB 2 tool provides a comprehensive framework for identifying risks of bias and it addresses several domains:

- Bias in randomization, evaluating the adequacy and documentation of participant randomization;
- Bias in adherence to intervention, assessing whether participants followed the assigned interventions as intended and accounted for deviations;
- Bias due to missing data, analyzing the impact of incomplete data and the appropriateness of handling methods;
- Bias in measurement of outcomes, determining the accuracy and reliability of outcome measurements and the potential influence of intervention awareness;
- Bias in the selection of reported results, assessing whether all pre-specified outcomes were reported and if selective reporting occurred.

The RoB-2 tool was implemented according to the guidelines of Sterne et al. (2019), ensuring a structured and transparent evaluation of methodological quality [13]. This process adheres to Cochrane standards for assessing bias in non-randomized studies.

### 2.4. Dealing with Missing Data

They were treated according to whether were ‘missing at random’ or ’not missing at random’. In relation to those missing at random, we analyzed available data and ignored missing data. For studies that reported a mean difference but no standard deviation, we used imputation [14].

### 2.5. Possible Risks of Heterogenicity

Risks of heterogenicity could be attributed to the study designs, the statistics adopted, the different populations or the clinical interventions used.

## 3. Results

### 3.1. Search Results

The process of article selection began with the application of the previously described inclusion and exclusion criteria, resulting in the retrieval of articles from the following databases: 144 from PubMed, 536 from the Cochrane Library, 42 from ClinicalTrials.gov, and 9 from Web of Science (WoS). Three investigators independently screened the titles and abstracts, resolving any disagreements through discussion and comparison of results. Duplicate records were removed (*n* = 130).

**Figure 1:**
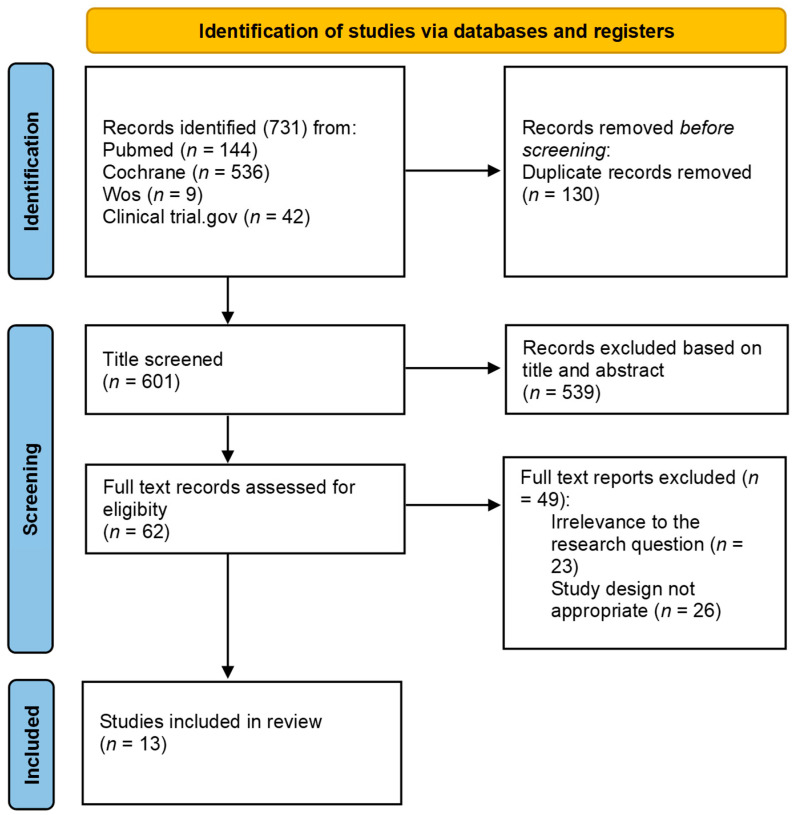
Flow chart of the analyzed studies.

Following the abstract screening, 62 articles remained and underwent a full-text review to assess their eligibility. Ultimately, 13 research articles fulfilled all the criteria and were included in the final review (Figure 1).

The methodological quality of the included studies was assessed using the modified Jadad Quality Scale. The scores assigned to each study, along with the evaluation of specific criteria, are presented in Table 1.

In line with Cochrane recommendations, the Risk-of-Bias 2 (RoB 2) tool was applied to systematically evaluate potential biases across several domains. 

This process ensures a structured and transparent evaluation of methodological quality, adhering to Cochrane standards.

**Figure 2:**
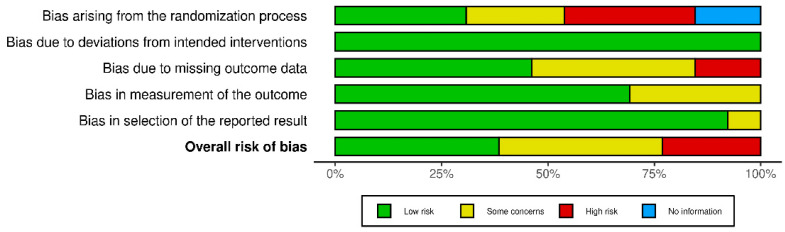
Summary of risk of bias across clinical studies. This image provides a visual summary of the risk of bias across various domains in the selected clinical studies. Each bar represents the percentage of studies categorized by their risk level—low-risk (green), some concerns (yellow), high-risk (red), and no information (blue)—for different bias domains, including randomization, intervention adherence, missing data, outcome measurement, and result selection.

**Figure 3:**
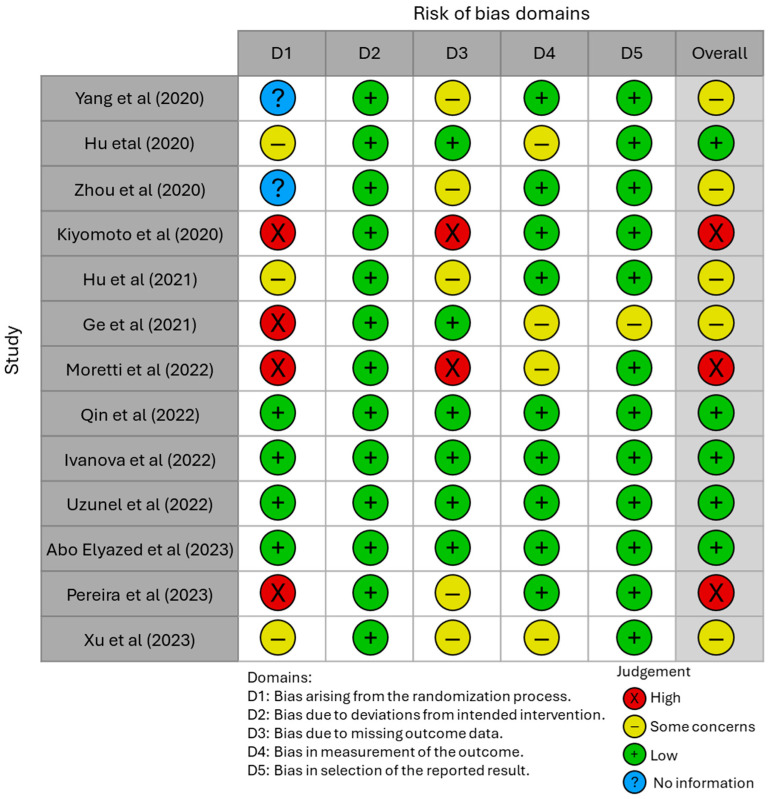
Risk of bias assessment of the selected studies. The table presents a detailed risk-of-bias assessment for each study, highlighting domain-specific evaluations for individual studies. The works cited in the table correspond to references [15, 16, 17, 18, 19, 20, 21, 22, 23, 24, 25, 26 and 27] in sequential order.

As recommended by Cochrane for the evaluation of risk of bias, an Excel graph was used (Figure 2). It allowed us to highlight the transparency and methodological rigor of the studies, and such an evaluation was performed for each included study (Figure 3).

Additionally, the domain-specific risk-of-bias evaluation for each study is detailed in Figure 3. 

The results of these assessments reveal that some studies achieved the highest Jadad scores and demonstrated low risk of bias across all domains. However, others exhibited some concerns or high risk in specific domains, underscoring the variability in methodological quality and rigor among the included studies. Despite this variability, the selected studies provide significant insights into recent advancements in understanding and treating the biochemical mechanisms underlying OP-related pain. They also highlight novel therapeutic and rehabilitation strategies for effectively managing this type of pain, contributing to a growing body of evidence supporting innovative approaches in this field (Table 2).

Xu et al. (2023) illustrated that exosomes secreted by myoblasts under mechanical stimulation can enhance osteogenesis in vitro and mitigate glucocorticoid-induced OP in vivo by targeting miR-92a-3p through the PTEN/AKT signaling pathway [27]. Ge et al. (2021) demonstrated that an Mg-MOF-based compound effectively downregulates gene expressions related to pain while upregulating the expression of osteogenic cytokines [20]. Ivanova et al. (2022) suggested that high levels of sclerostin, associated with lower bone mineral density (BMD) in patients with Gaucher disease, involve the Wnt/β-catenin pathway in bone pathology [23]. Similarly, Dai et al. (2023) discussed the effectiveness of sclerostin monoclonal antibodies in enhancing BMD among postmenopausal women with OP [28]. 

Taken together, these findings provide a comprehensive view of various molecular mechanisms implicated in bone health and identify potential therapeutic targets for OP. The following sections will delve into these mechanisms and their implications.

### 3.2. Emerging Insights into Pathophysiological Mechanisms in Osteoporosis and OP-Related Pain

**Figure 4:**
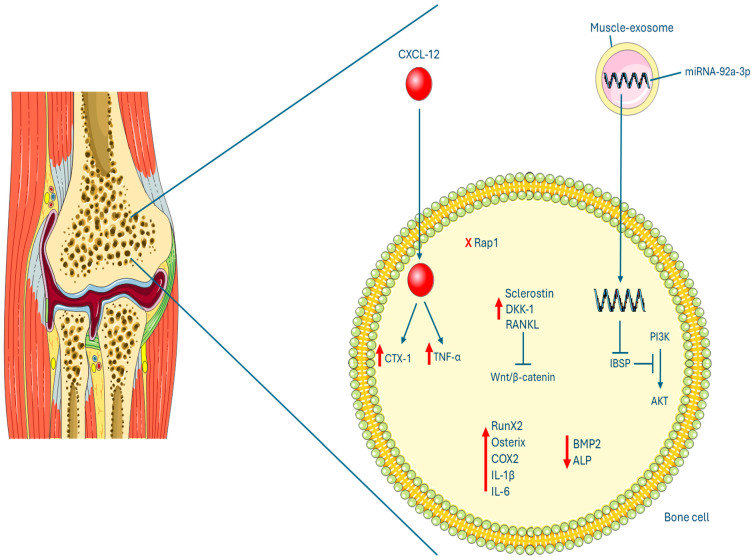
Graphical representation of dysregulated pathways in OP. Arrows denote higher expression, while “x” indicates disruption of the pathway.

OP is a debilitating condition characterized by the progressive loss of bone mass and strength. Recent research has revealed complex connections between skeletal muscle and bone, offering new insights into the regulatory pathways that control bone metabolism and, consequently, OP-related pain. The decline in bone mass and strength in OP is driven by a range of biochemical and neurophysiological changes (Figure 4). A significant contributor is the disruption of mechanical coupling between skeletal muscle and bone [29]. Muscle contractions generate mechanical stimuli transmitted to bone, thereby eliciting responses from osteocytes and osteoblasts to foster bone formation [30]. This mechanotransduction pathway is compromised in OP, precipitating a decline in bone mass and strength [31,32].

Besides mechanical interactions, muscle and bone engage in reciprocal communication via endocrine and paracrine signaling [33]. Muscle-derived exosomes, especially those induced by mechanical stimuli, have been demonstrated to enhance the proliferation and osteogenic differentiation of BMSCs [27]. This process involves the upregulation of miR-92a-3p within these exosomes, which targets the PTEN protein and activates the PI3K/AKT signaling pathway, thereby promoting osteogenesis [27]. The PI3K/AKT pathway serves as a central regulator of various cellular processes, including cell growth, survival, proliferation, and differentiation [34]. 

Targeting the miR-92a-3p/PTEN/AKT signaling axis offers a promising avenue for OP treatment and bone regeneration [27]. In this sense, a study by Hu et al. (2021) observed a significant increase in miRNA-92a-3p expression in patients with concurrent fractures and brain trauma, as well as in a murine model [19]. miRNA-92a-3p was found to facilitate the mineralization and maturation of osteoblast precursor cells in vitro [19]. Mechanistically, miRNA-92a-3p targets and suppresses the expression of IBSP (osteopontin), a protein highly expressed in osteoblasts, osteoclasts, and chondrocytes, consequently upregulating osteogenesis-related gene expression and promoting bone formation. This process is mediated through the activation of the PI3K/AKT signaling pathway, which plays a crucial role in regulating osteogenic precursor cell differentiation and activity.

Patients with concurrent fractures and brain trauma exhibited accelerated callus formation and more efficient fracture healing compared to those with isolated fractures, which was attributable to the upregulation of miRNA-92a-3p expression and the subsequent enhancement of osteoblast maturation and bone formation via the described signaling pathway [19]. However, the researchers acknowledged limitations in their clinical data collection, including variations in callus sampling time among patients, which could have influenced the expression-level disparities.

In summary, the investigation identified a novel mechanism involving miRNA-92a-3p modulation of the IBSP-PI3K/AKT axis, elucidating the improved fracture healing observed in patients with concurrent brain trauma [19]. 

Moreover, recent research highlighted an inverse relationship between elevated plasma levels of the chemokine CXCL12 and bone mineral density (BMD) at various skeletal sites in patients with postmenopausal OP (PMOP) [15]. Increased CXCL12 levels positively correlated with the inflammatory marker TNF-α and the bone resorption marker CTX-1, suggesting that CXCL12 may promote osteoclast activity and bone loss [15]. Additionally, the CXCL12/C-X-C chemokine receptor type 4 (CXCR4) signaling axis was implicated in the pathogenesis of pain and functional impairment in PMOP patients. Specifically, CXCL12 and its receptor CXCR4 were found to be involved in the peripheral and central sensitization processes that contribute to osteoporotic pain.

Importantly, elevated plasma CXCL12 levels were associated with greater disease severity in PMOP. This was highlighted by lower BMD, as well as higher scores on pain (VAS) and disability (ODI) assessments [15].

Taken together, these findings indicate that increased systemic CXCL12 expression may serve as a candidate biomarker to reflect disease progression and severity in PMOP. However, the cross-sectional nature of the study limits the ability to establish causal relationships between CXCL12 and clinical outcomes. Longitudinal studies will be needed to validate the predictive value of CXCL12 as a biomarker in PMOP [15].

Additionally, in postmenopausal OP, dysregulation of the calcium-sensing receptor has been identified, resulting in inadequate calcium homeostasis and enhanced bone loss in osteoblasts, osteoclasts, and osteocytes [17]. This dysregulation contributes to increased bone loss and decreased bone density, leading to a higher risk of fractures. Furthermore, the impairment of the cGMP-PKG signaling pathway, specifically involving the phosphatidylinositol 3-kinase/Akt/endothelial nitric oxide synthase/nitric oxide/cGMP/PKG cascade, disrupts osteogenesis in bone marrow mesenchymal stem cells, resulting in reduced osteogenesis and impaired bone formation. Additionally, altered endocytosis of bisphosphonates, which are commonly used in OP treatment, adversely affects osteoclast function, leading to an altered response to bisphosphonate treatment [17].

Dysregulation of the Rap1 signaling pathway, particularly in the Rap1-mediated facilitation of talin/integrin interaction, results in compromised osteoclast activity and disrupts the bone remodeling process by affecting both osteoblast and osteoclast function. Moreover, disruption of the AMPK signaling pathway, which plays a critical role in osteoblastic differentiation, is closely associated with the pathophysiology of postmenopausal OP. These combined factors contribute to an increased risk of developing OP-related complications, such as fractures, in postmenopausal women [17].

Furthermore, it has been demonstrated that several genes, including *Plekha2*, *Plekhb1*, *Pnpla7*, *Scd*, *Mgst3*, *Tsnax*, *Prkcz*, *Gna11*, *Col4a1*, *Sox6*, *Ace*, *Syk*, and *Tgfb3,* are differentially methylated and associated with the altered signaling pathways, suggesting their potential roles in the pathogenesis of PMOP [17].

As described above, OP is characterized by weakened bone density and structure, leading to an increased risk of fractures. A key driver of the pain experienced by patients with OP is the alteration of signaling pathways involved in pain perception and transmission.

Another signaling pathways affected in OP is the Rank–Rankl–osteoprotegerin (OPG) pathway. In healthy bone, this pathway regulates the balance between osteoclasts (cells that break down bone) and osteoblasts (cells that build bone). However, in OP, the Rank–Rankl–OPG pathway becomes dysregulated, leading to increased osteoclast activity and excessive bone resorption [22].

This imbalance not only weakens the bones but also leads to the sensitization of pain receptors within the bone. The increased activity of osteoclasts and the subsequent release of inflammatory mediators, such as prostaglandins and cytokines, can directly stimulate and sensitize nociceptors (pain-sensing nerve fibers) in the bone, causing chronic pain and discomfort for patients [22].

Furthermore, the structural changes in the bone, such as microfractures and loss of trabecular architecture, can also contribute to the pain experienced by individuals with OP. These alterations in bone structure can lead to increased mechanical stress on the remaining bone, further activating pain pathways and resulting in a heightened pain response [26]. Patients with OP often report a range of pain symptoms, including constant dull aches, sharp shooting pains, and increased sensitivity to movement or pressure. The severity of the pain can vary depending on the stage of the disease, the presence of fractures, and the individual’s response to the underlying pathophysiological changes [22]. Understanding the alterations in signaling pathways and the resulting pain experiences is crucial for the effective management of OP-related pain. Targeted therapies that address the dysregulation of the Rank–Rankl–OPG pathway, as well as pain management strategies, can help improve the quality of life of individuals suffering from the chronic pain associated with OP [22].

In a study by Uzunel et al. (2023), no significant differences were found in the plasma levels of the sensory neuropeptides SP and calcitonin gene-related peptide among spinal orthosis, control, and exercise groups [24]. In fact, the use of a spinal orthosis was associated with lower levels of inflammatory IL-6 at 6 months compared to the control and exercise groups. This suggests that the use of a spinal orthosis may have had an effect on the inflammatory signaling pathway, but not on the sensory neuropeptide pathways, in the management of OP-related pain [24]. 

In a randomized controlled trial, High-Level Laser Therapy (HILT) and extracorporeal shockwave therapy (ESWT) were used in combination with traditional physical therapy to treat osteoporotic long-term hemiparetic patients suffering from OP-related pain [25]. HILT was found to be more effective than ESWT in terms of improving overall stability, balance, quality of life, and reducing pain (as measured by VAS scores). The proposed mechanisms include that HILT could cause an increase in mitochondrial oxidative reactions, ATP/DNA/RNA production, and reduced thermal buildup in deep tissues, leading to improved tissue function and repair [25]. At the same time, it was proposed that ESWT enhances neovascularization and upregulates osteogenic/angiogenic growth factors (proliferating cell nuclear antigen PCNA, eNOS, BMP-2, VEGF) at bone–tendon junctions in order to promote bone and vascular remodeling [25,35]. In this sense, combining HILT and ESWT with traditional physical therapy was more effective than physical therapy alone when it came to improving overall stability index and balance (SFBBS), increasing quality of life (QUALEFFO-41 questionnaire) and reducing pain (VAS scores) [25]. Additionally, HILT was found to be superior to ESWT for most outcomes, except for VAS pain scores.

The improved stability, balance, quality of life, and reduced pain likely had a positive impact on the stroke patients’ activities of daily living and overall functional status [25]. 

In IJO, researchers identified elevated levels of sclerostin in osteocytes, which were particularly pronounced in IJO patients compared to healthy controls. This increased sclerostin expression correlates with heightened markers of Wnt signaling inhibition within these osteocytes. Since Wnt signaling is crucial for bone formation mediated by osteoblasts, this inhibition is believed to hinder bone formation in IJO patients. Consequently, this disruption in the sclerostin–Wnt signaling axis likely contributes to the reduced bone mass and compromised bone strength observed in these individuals [26]. The decreased bone formation and weakened bone structure of IJO patients elevate their susceptibility to fragility fractures. Moreover, the dysregulation of sclerostin and Wnt signaling during critical growth phases in children with IJO may impair skeletal development and maturation. Left untreated, progressive bone loss and skeletal fragility in IJO could lead to significant long-term consequences, including increased morbidity and mortality due to fractures and associated complications [26]. These insights suggest that targeting the sclerostin–Wnt signaling pathway could represent a promising therapeutic approach for managing IJO and other pediatric bone disorders characterized by reduced bone formation. Such interventions could potentially mitigate the adverse impacts on bone mass, strength, and overall skeletal development in affected individuals [26]. In a related study, sclerostin’s role as a potential biomarker for Gaucher Disease (GD) was investigated, with particular focus on its influence on the Wnt/β-catenin signaling pathway [23]. This research highlighted sclerostin fluctuating levels and interactions with other regulatory factors within this pathway in GD patients. Elevated sclerostin levels were notably associated with symptoms such as bone pain, bone marrow infiltration, and EM flask deformity in GD patients as they aged [23]. The study also explored the relationship between sclerostin and DKK-1, another inhibitor of the Wnt/β-catenin pathway, noting significant differences in their ratio in GD patients with and without bone pain. Additionally, the research examined the link between DKK-1 and Rankl, finding varying correlations among different GD patient groups [23]. Furthermore, the study investigated β-catenin and Wnt5a serum levels, which are involved in osteoclast formation and non-canonical Wnt signaling. It was found that these levels did not significantly differ between GD patients and controls, suggesting a limited impact on the observed metabolic changes [23]. 

A recent study allows us to understand that in osteoporotic patients with chronic back pain, nociceptive pain predominates, but there is also a significant neuropathic component. The emergence of neuropathic pain in osteoporotic vertebral fractures (OVFs) is associated with chronic inflammation, mechanical alterations, and central sensitization. These pathways significantly disrupt metabolic processes involving inflammation, spinal biomechanical changes, and persistent nociceptive stimuli, triggering peripheral and central sensitization [21]. Specifically, this cross-sectional study conducted by Moretti et al. (2022) showed that among the 72 patients enrolled—mostly women (88.8%) with an average age of 69.2 ± 8.9 years—a majority (70.8%) had multiple vertebral fractures, primarily in the thoracic spine. Pain severity was generally mild, with mean BPI severity and interference scores of 4.0 ± 1.8 and 4.3 ± 2.1, respectively. The painDETECT questionnaire (PD-Q) indicated that 82% experienced nociceptive pain, whereas only 5.5% exhibited neuropathic pain. However, the Leeds assessment of neuropathic symptoms and signs pain scale (LANSS) identified a likely neuropathic mechanism in 23.6% of cases [21]. The study highlighted a notable disparity between LANSS and PD-Q scales in identifying neuropathic pain. The LANSS, incorporating physical examination for signs like allodynia and hyperalgesia, detected a higher incidence of neuropathic pain, contrasting with the PD-Q, which tended to categorize pain as “mixed,” resulting in a lower neuropathic pain detection rate [21]. Regarding OVF location, single thoracic fractures correlated with a higher prevalence of neuropathic pain, whereas the PD-Q identified increased neuropathic pain prevalence among patients with multiple lumbar OVFs. Notably, no significant correlation was found between the Spine Deformity Index (SDI) and the pain type (nociceptive vs. neuropathic). Surprisingly, despite varying numbers and locations of OVFs, patients reported mild pain and minimal interference with daily activities. This outcome may be attributed to the early initiation of anti-osteoporotic drug therapy within a specialized setting such as the Fracture Liaison Service, potentially contributing to the reduced pain severity and interference observed [21]. 

The latest research has highlighted exacerbated bone resorption and altered bone micro-architecture in osteoporotic conditions, which further intensified pain-like behaviors [18]. The study investigated the impact of ovariectomy (OVX) and monosodium iodoacetate (MIA) injection on osteoarthritis (OA) and OP in C57BL/6 J mice, focusing on cartilage degeneration, bone structure, pain-related behaviors, and the expression of key regulators in bone formation and resorption [18]. 

Histological analysis revealed significant cartilage damage in OA mice, regardless of OVX status, with high Osteoarthritis Research Society International (OARSI) scores persisting up to 10 weeks post-MIA injection. Synovitis was notably elevated in OA mice at 2 weeks, but no differences were observed at 6 and 10 weeks compared to controls and OP groups. Serum TRAP5b levels, indicative of bone resorption, were significantly higher in mice with OP and OA-OP compared to controls and OA-only mice, suggesting increased bone resorption activity in osteoporotic conditions. Micro-computed tomography (μCT) analysis showed lower bone volume ratio (BS/BV), bone volume fraction (BV/TV), and trabecular number (Tb.N), and higher trabecular separation (Tb.Sp) in OP and OA-OP mice, indicating compromised bone structure. Expression levels of Runt-related transcription factor 2 (Runx2), Osterix, and Rankl were significantly elevated in these groups, reflecting enhanced osteoblast and osteoclast differentiation regulation [18]. Treatment with alendronate (ALN), a medication for OP, significantly reduced osteoclast numbers and expression levels of Runx2, Osterix, and Rankl in OA-OP mice. ALN also improved pain-related behaviors, lasting over four weeks after the treatment, as demonstrated by von Frey and paw-flick tests. In contrast, carprofen, a nonsteroidal anti-inflammatory drug, provided partial and temporary pain relief during administration only. Mice with OA and OP exhibited prolonged and intensified pain-related behaviors compared to those with OA or OP alone, and these behaviors were alleviated significantly by ALN treatment, aligning their responses with those of control mice [18]. Complementary to these therapeutic approaches, recent reviews and studies have also explored the clinical implications of hyaluronic acid in musculoskeletal rehabilitation, underscoring its role in joint health and recovery [36,37]. Moreover, the modulation of OA-related cytokine profiles through the use of terpenes has been examined, revealing significant potential in reducing inflammation and improving patient outcomes [38,39]. These insights, together with clinical approaches to cartilage damage and the utilization of nutraceuticals in knee OA, represent a multifaceted strategy in addressing the complex interplay of OA and OP [40,41,42].

Another study that evaluated the therapeutic potential of Ket@Mg-MOF-74 as a drug delivery system for OP highlighted its effectiveness in treating the condition by alleviating pain, suppressing inflammation, and promoting bone formation. These effects were achieved through the synergistic actions of ketoprofen and magnesium ions released from the MOF structure [20]. This study showed reductions in COX2 expression and enhanced expression of osteogenic markers (BMP2, Runx2, ALP) after the treatment with Ket@Mg-MOF-74 [20]. Additionally, inflammatory cytokine expression (TNF-α, IL-1β, IL-6) was notably decreased, suggesting some enhanced anti-inflammatory effects facilitated by ketoprofen and magnesium ions released from the MOF [20].

In their study, Hu et al. (2020) investigated the potential benefits of zoledronic acid (ZOL) for patients suffering from osteoporotic vertebral fractures. ZOL acts by slowing down the breakdown of bone, which is a critical factor in maintaining bone strength and density. Their findings revealed compelling insights into its therapeutic effects [16]. The researchers observed a significant decrease in the levels of two important markers of bone turnover among patients receiving ZOL treatment: P1NP, which signifies bone formation, and β-CTX, which indicates bone breakdown. This reduction suggests that ZOL effectively inhibited processes that contribute to bone loss, thereby influencing both bone formation and resorption [16]. Additionally, the study showed that patients treated with ZOL exhibited higher bone mineral density (BMD) in the femoral neck area compared to those who did not receive the treatment, both at the 6-month and at 12-month follow-up evaluations. This BMD increase suggests that ZOL contributed to maintaining or possibly improving bone density over time, potentially by reducing the activity of cells that break down bone and promoting mineralization processes [16]. From a functional standpoint, patients treated with ZOL reported significant improvements in pain relief and functional outcomes. These improvements were quantified using standardized tools such as the VAS for pain assessment and the ODI for functional impairment. These findings indicate that ZOL not only supports bone health but also contributes to overall recovery and reduces pain in individuals suffering from osteoporotic vertebral fractures [16]. 

These results reveal the complex relationships between bones and muscles and offer new insights into how the body regulates bone metabolism. OP, which is characterized by weakened bones due to reduced density, arises from changes in both biochemical processes and nerve–muscle interactions, which weaken the critical connections between them and cause acute and chronic pain. Specific pathways, including miR-NA-92a-3p/PTEN/AKT, might allow available therapies to accelerate bone healing and prevent fractures at an earlier stage, but also to prevent and relieve OP-related pain. Such biomarkers could play a crucial role in understanding and managing OP in postmenopausal women, particularly by focusing on CXCL12. Tailoring treatment to these pathways may reduce pain and enhance the quality of life of OP patients.

### 3.3. Rehabilitation and Therapeutic Strategies for OP-Related Pain Management

Managing OP involves a range of treatments and rehabilitative approaches aimed at reducing pain and improving patients’ functional outcomes. These therapeutic strategies are based on the knowledge of the pathophysiological mechanisms described above, and they have the goal of counteracting the mentioned biochemical and biomechanical factors which underlie OP and the pain it determines, especially chronic pain. 

Nonpharmacological therapies, including physical exercise, have gained prominence for their role in maintaining or increasing bone mass and their broader benefits in pain management [43]. Physical exercise is central to OP rehabilitation, shifting from a primary focus on bone mass preservation to a holistic approach that improves overall function and reduces chronic pain [44]. Complementary approaches such as yoga and Pilates are now recognized for their positive impact on pain and function in OP patients. In fact, yoga has been shown to reduce pain levels, while Pilates can increase BMD, enhance quality of life, and consequently relieve pain [45]. Also, the Wuqinxi-based exercise program, when conducted for at least six months, provides significant pain relief [46]. Obviously, it is crucial that exercises be performed safely, with gradual progression and careful avoidance of trunk bending and rotation to prevent exacerbating back pain. The standard rehabilitation approach for OP patients includes correcting postural changes, preventing falls by improving balance and coordination, relieving pain, and enhancing psychological well-being. Group rehabilitation sessions are often recommended to teach exercises, enabling patients to practice independently at home [43]. 

Power training has been noted to be particularly effective in maintaining BMD compared to strength training. Exercises designed to improve axial stability, such as back extension programs, should be tailored to the patient’s needs. A comprehensive exercise regimen should target flexibility, muscle strength, core stability, cardiovascular fitness, and gait steadiness [47]. Postural taping, which involves applying tape to the skin to enhance proprioceptive feedback and improve thoracic extension, has been shown to lower pain in OP patients. Additionally, postural deformities and muscle contractures in OP can benefit from soft manual tissue massage therapy combined with exercises [48,49]. Physical therapy methods also include magnetic fields and vibration training, which are similarly useful for managing OP-related pain [50]. Although evidence on magnetic fields is limited, low-frequency pulsed electromagnetic fields (PEMFs) have been reported to provide quick and effective relief from primary OP pain [50]. Vibration training enhances muscle strength, neuromuscular coordination, and balance, thereby reducing the risk of falls and fractures. Despite its benefits, the optimal vibration frequency for therapeutic effects remains unclear, and therapy should start at lower frequencies and increase gradually [50]. 

Nonpharmacological therapies such as cognitive behavioral therapy (CBT) and mindfulness-based interventions offer additional benefits for managing chronic pain. CBT helps patients understand their pain and develop coping strategies, improving pain management and quality of life. Mindfulness-based interventions, through meditation and mindful awareness, assist patients in managing pain by viewing it as a series of discrete events rather than a persistent problem [51]. 

The efficacy of percutaneous vertebroplasty (PV) in treating osteoporotic vertebral compression fractures (OVCFs) has been debated. The VERTOS V trial by Carli et al. (2023) compared PV with active controls in patients with chronic painful OVCFs. The results showed a significant pain reduction in the PV group, with lower VAS scores at 3 and 12 months (PV: 3.5 vs. control: 4.9 at 3 months, PV: 3.9 vs. control: 5.1 at 12 months). Over 12 months, PV patients had a greater reduction in VAS scores (PV: 3.6 vs. control: 2.3) and better quality of life improvements (PV: 7.5 vs. control: 2.3). However, no significant differences were seen in Roland Morris Disability Questionnaire scores. PV showed safety regarding new fractures and vertebral height loss, but the study’s small sample size and active control design warrant caution. Larger multicenter trials with sham controls are needed to confirm its efficacy and safety [52]. 

Although the superiority of this procedure over conservative treatments is debated, it has demonstrated better outcomes in pain relief and function for women aged ≥ 60 years with OP [53]. Hip fractures, prevalent in OP patients, often require hip arthroplasty, with cemented and uncemented femoral implants yielding good results. However, uncemented implants may have a higher incidence of periprosthetic fractures, and both types face risks of dislocation [54]. 

Advancements in surgical techniques are another crucial aspect of OP treatment. Shi et al. (2023) compared deflectable percutaneous kyphoplasty (DPKP) with conventional bilateral percutaneous kyphoplasty (BPKP) for OVCFs. Their findings indicated that DPKP reduced operative time, improved VAS and ODI scores, and better restored kyphosis angle and vertebral height, with fewer fluoroscopy instances and lower cement leakage rates compared to BPKP [55]. Similarly, Hong et al. (2023) highlighted the benefits of balloon kyphoplasty via a unilaterally extrapedicular approach in lumbar OVCFs, noting superior initial pain relief and more symmetric cement distribution compared to conventional methods [56]. 

Beyond this, complementary and rehabilitative approaches play a critical role in managing OP. 

Physical energies are also crucial in managing pain and improving function in OP patients. Abo Elyazed et al. (2023) evaluated the combination of HILT and extracorporeal shockwave therapy (ESWT) with traditional physical therapy for osteoporotic long-term hemiparetic patients. Both HILT and ESWT effectively reduced VAS pain scores compared to traditional physical therapy alone, with HILT showing superior efficacy in pain reduction. These therapies also enhanced stability and promoted osteogenic and angiogenic factors, with HILT providing slight advantages when it came to improving quality-of-life metrics. The study highlighted the need for further research to explore gender-specific treatment responses and validate these findings across diverse populations [25]. 

Pharmacological therapy is a fundamental approach to managing chronic pain, especially when nonpharmacological methods fall short. The treatment should be adjusted based on the patient’s reported pain intensity. For mild pain, nonsteroidal anti-inflammatory drugs (NSAIDs) or acetaminophen are typically recommended. For moderate pain, weak opioids may be combined with NSAIDs or acetaminophen. Severe pain often requires stronger opioids. While adjuvant drugs can improve pain relief, they should be used cautiously due to potential side effects, abuse, and addiction risks [43]. Pharmacological options are often considered in conjunction with or following nonpharmacological therapies.

A multimodal approach, integrating both pharmacological and nonpharmacological treatments, is particularly effective for persistent and resistant pain. For patients with OP but no fractures, nonpharmacological therapies such as physical exercise, cognitive behavioral therapy, mindfulness-based pain management, and physical therapy are recommended. For those with fractures, a combination of pharmacological and nonpharmacological treatments is advised, with bisphosphonates and teriparatide showing promise in reducing pain and preventing new fractures [57]. 

Emerging pharmacological treatments have also shown promise in OP management. Dai et al. (2023) conducted a phase 1 trial on SHR-1222, a monoclonal antibody targeting sclerostin, in PMOP. The trial reported that SHR-1222 had a favorable safety profile and significantly increased BMD at various skeletal sites, particularly in the 300 mg once-monthly cohort, indicating the potential of new pharmacological agents to complement existing therapeutic strategies [28]. 

In conclusion, the management of pain-related OP requires a comprehensive approach that integrates advanced rehabilitation strategies and therapeutic interventions. Future research should aim to validate these interventions across diverse patient populations and explore their long-term outcomes to further refine clinical practices and enhance patients’ quality of life. By focusing on a multidisciplinary approach that includes surgical, pharmacological, and rehabilitative strategies, clinicians can optimize treatment outcomes for OP patients, particularly for the management of chronic pain associated with OVCFs.

## 4. Discussion

Managing OP requires a multifaceted approach that addresses both biochemical and biomechanical disruptions inherent to the disease. OP is marked by reduced bone mass and strength and compromised mechanical coupling between muscle and bone. Effective management, therefore, necessitates an integration of diverse treatment strategies, encompassing both nonpharmacological and pharmacological therapies, each targeting different aspects of patient care.

Recent research highlights the complex nature of OP, which arises from intricate biochemical and biomechanical disruptions. One critical aspect is the impaired mechanical coupling between muscle and bone, which affects bone formation and strength due to compromised mechanotransduction pathways. This underscores the importance of muscle–bone interactions in maintaining bone health and suggests that interventions aimed at improving mechanical loading and muscle strength could be beneficial.

A significant finding in this context is the role of miR-92a-3p in bone metabolism. This microRNA, present in muscle-derived exosomes, promotes osteogenic differentiation and proliferation by targeting the PTEN protein and activating the PI3K/AKT signaling pathway. Elevated levels of miR-92a-3p have been associated with improved bone healing in patients with fractures and brain trauma, indicating its potential as a therapeutic target. Modulating the miR-92a-3p/PTEN/AKT axis could therefore accelerate bone healing and prevent fractures, offering a promising avenue for OP treatment.

Moreover, elevated levels of CXCL12 have been linked to reduced BMD and increased inflammatory markers, suggesting its role in osteoclast activity and disease progression. While current cross-sectional studies limit causal conclusions, CXCL12’s potential as a biomarker warrants further investigation through longitudinal studies to confirm its predictive value. Additionally, disruptions in various signaling pathways, including calcium-sensing receptors, cGMP-PKG, Rap1, and AMPK, contribute to impaired osteogenesis and increased fracture risk in OP. These insights provide potential therapeutic targets and highlight the complexity of OP pathophysiology.

Pain management is another critical aspect of OP treatment. Bone is highly innervated, primarily by thinly myelinated sensory nerve fibers and Calcitonin Gene-Related Peptide (CGRP), with minimal innervation by larger, rapidly conducting fibers. As bone mass and strength decline with age, the density of sensory nerve fibers in bone tissue increases, leading to heightened sensitivity and pain in older individuals. This increase in sensory innervation, combined with nociceptor sensitization due to osteoclastic activity, significantly contributes to bone pain in OP [58,59]. 

Nonpharmacological treatments, particularly physical exercise, have become central to OP rehabilitation. Exercise not only helps maintain or increase bone mass but also improves functional outcomes and pain management. Various exercise modalities, such as yoga and Pilates, have shown positive effects on pain relief and BMD. Yoga helps with pain reduction, while Pilates enhances BMD, quality of life, and pain management [60]. Similarly, Wuqinxi-based exercise programs have proven effective in relieving pain. However, exercises should be performed safely, with gradual progression and avoidance of activities that might exacerbate back pain.

Power training, in contrast to strength training, focuses on developing explosive strength and movement speed by using lighter weights and faster movements, while strength training aims to increase maximal strength through heavier loads and slower, more controlled movements. Both approaches have been shown to be effective in improving bone mineral density and muscle strength [61]. A comprehensive exercise regimen should include flexibility, muscle strength, core stability, cardiovascular fitness, and gait steadiness. Additional techniques like postural taping and soft manual tissue massage therapy can also benefit OP patients by improving proprioceptive feedback, relieving pain, and addressing postural deformities and muscle contractures.

Emerging therapies, such as magnetic fields and vibration training, show promise [50]. Indeed vibration training enhances muscle strength, neuromuscular coordination, and balance, though the optimal frequency for therapeutic effects remains uncertain and should start at lower frequencies, increasing gradually [50].

When nonpharmacological methods are insufficient, pharmacological therapies are essential. Mild pain often responds to NSAIDs or acetaminophen, while moderate pain may require weak opioids combined with NSAIDs or acetaminophen [43]. Severe pain might necessitate stronger opioids, which should be administered with caution to avoid side effects and abuse risks [43].

Recent advancements in pharmacological treatments (SHR-1222) show promise. This agent has demonstrated a favorable safety profile and significantly increased BMD at various skeletal sites, highlighting its potential to complement existing therapies [28].

Surgical interventions are employed to address vertebral fractures resulting from OP. While the efficacy of PV compared to conservative treatments is debated, studies like the VERTOS V trial have shown significant pain reduction and quality-of-life improvements in patients treated with PV [52]. However, the small sample sizes in these studies highlight the need for larger multicenter trials to validate these findings.

In conclusion, a comprehensive approach to OP management integrates nonpharmacological and pharmacological therapies. Nonpharmacological strategies, including exercise, CBT, and mindfulness-based interventions, offer valuable benefits for managing chronic pain and improving quality of life.

Overall, these advancements in the biochemical and clinical understanding of OP not only enhance our comprehension of the disease but also open up new possibilities for innovative therapies and personalized treatments. Improved insights into the mechanisms of pain in OP offer the potential for more effective pain management strategies, ultimately enhancing patient outcomes and quality of life. 

This systematic review acknowledges a couple of limitations. First, the use of the “text availability: full text” filter during the search process may have resulted in the exclusion of some relevant studies that were only available in abstract or partial form. While this approach ensured the inclusion of complete and verifiable data, it is possible that some valuable insights were not captured.

Moreover, there is variability in the methodological quality of the included studies. Eight studies had scores below three, and three studies showed a higher risk of bias. These factors should be considered when interpreting the findings, as they may influence the robustness and generalizability of the conclusions.

Future research should address existing methodological limitations and continue to explore the integration of biochemical and clinical approaches to refine and advance OP management.

## 5. Conclusions

This systematic review highlights significant advancements in understanding OP pathophysiology, emphasizing molecular mechanisms related to bone metabolism and pain. OP is characterized by heterogeneous loss of bone mass and strength, with detailed insights into the biochemical and neurophysiological changes contributing to this disease. Key findings include the impact of skeletal muscle disconnection on bone development and the importance of mechanotransduction in bone development. These insights open up promising avenues for targeted therapies aimed at enhancing bone formation and alleviating pain.

Nonpharmacological treatments, such as exercise and innovative therapies like magnetic fields and vibration training, have shown potential in improving patient outcomes. Pharmacological and surgical interventions also play vital roles in managing OP and related pain.

However, this review acknowledges the need for further research to address methodological limitations and fully explore the potential of molecular biomarkers in OP management. 

Future studies should aim to refine these therapeutic strategies and enhance their applicability in clinical practice, ultimately improving patients’ quality of life and disease management.

## Figures and Tables

**Table 1:** The modified version of Jadad quality scale.

Authors and Refs.	Was the Treatment Randomly Allocated?	Was the Randomization Procedure Described and Was Appropriate?	Was There a Description of Withdrawals and Dropout?	Was There a Clear Description of the Inclusion/Exclusion Criteria?	Were the Methods of Statistical Analysis Described?	Jadad Score (0–5)
Yang et al., 2020 [15]	No	No	No	Yes	Yes	2
Hu et al., 2020 [16]	Yes	No	Yes	Yes	Yes	4
Zhou et al., 2020 [17]	No	No	No	Yes	Yes	2
Kiyomoto et al., 2020 [18]	No	No	No	Yes	Yes	2
Hu et al., 2021 [19]	No	No	No	Yes	Yes	2
Ge et al., 2021 [20]	No	No	No	No	Yes	1
Moretti et al., 2022 [21]	No	No	No	Yes	Yes	2
Qin et al., 2022 [22]	Yes	No	Yes	Yes	Yes	4
Ivanova et al., 2022 [23]	Yes	Yes	Yes	Yes	Yes	5
Uzunel et al., 2022 [24]	Yes	Yes	Yes	Yes	Yes	5
Abo Elyazed et al., 2023 [25]	Yes	Yes	Yes	Yes	Yes	5
Pereira et al., 2023 [26]	No	No	No	Yes	Yes	2
Xu et al., 2023 [27]	No	No	No	Yes	Yes	2

**Table 2:** Selected studies on biochemical mechanisms and therapeutic strategies in osteoporosis and related pain.

Title and Refs.	Sample	Measure	Results	Pathway and Therapeutic Strategy
Elevated plasma C-X-C motif chemokine ligand 12 (CXCL12)/stromal cell-derived factor 1 (SDF-1) levels are linked with severity of postmenopausal osteoporosis [15]	91 patients with postmenopausal OP (PMOP)	Plasma CXCL12 levels, BMD (g/cm^2^) evaluated by DXA, VAS, ODI, CTX-1, tumor necrosis factor α (TNF-α)	- Elevated CXCL12 levels in PMOP patients vs. postmenopausal non-osteoporotic (PMNOP) and healthy controls - Higher plasma CXCL12 levels in patients with vertebral fractures - Negative correlation between CXCL12 levels and BMD at femoral neck, total hip, and lumbar spine - Positive correlation of CXCL12 levels with clinical severity measured by VAS and ODI - Positive association with CTX-1 and TNF-α levels	Monitoring plasma CXCL12 levels to evaluate disease severity and guide therapeutic targeting through CXCL12/SDF-1 signaling in PMOP patients
Effect of preoperative zoledronic acid administration on pain intensity after percutaneous vertebroplasty for osteoporotic vertebral compression fractures [16]	242 patients with OVCF diagnosis (125 men, 117 women)	Pain (VAS), functional activity (ODI), bone mineral density (BMD), serum markers (P1NP, β-CTX)	- ZOL group showed significant improvements in VAS, ODI and BMD - Reduced serum markers - Fewer new fractures (2 vs. 13 in control) - Similar complication rates between the 2 groups	Pre-surgery ZOL administration effectively reduces pain, improves function, increases BMD and decreases fracture risk with manageable adverse effects
Alterations in DNA methylation profiles in cancellous bone of postmenopausal women with osteoporosis [17]	5 postmenopausal women with osteoporosis and 3 healthy controls	DNA methylation profiles using Illumina 850K methylation microarray	- Identified 13 differentially methylated genes: pleckstrin homology domain containing A2 (*Plekha2*), *Plekhb1*, patatin-like phospholipase domain-containing 7 (*Pnpla7*), stearoyl-CoA desaturase (*Scd*), Microsomal glutathione S-transferase 3 (*Mgst3*), translin-associated factor X (*Tsnax*), protein kinase C zeta (*Prkcz*), G protein subunit alpha 11 (*Gna11*), collagen type IV alpha 1 chain (*Col4a1*), SRY-Box Transcription Factor 6 (*Sox6*), angiotensin I-converting enzyme (*Ace*), spleen-associated tyrosine kinase (*Syk*), transforming growth factor beta 3 (*Tgfb3*). - 9603 hypermethylated sites and 5706 hypomethylated sites. - Significant methylation differences found in promoter regions and CpG islands. - Enriched pathways: calcium signaling, (Guanosine 3′,5′-cyclic monophosphate-dependent protein kinase G) cGMP-PKG signaling, endocytosis, Ras-related protein 1 (Rap1), adenosine monophosphate-activated protein kinase (AMPK). - Upregulation of SCD, MGST3, TSNAX, TGFB3 and downregulation of SOX6, GNA11	Involvement of calcium signaling, cGMP-PKG, endocytosis, Rap1, AMPK pathways with significant gene methylation changes
High bone turnover state under osteoporotic changes induces pain-like behaviors in mild osteoarthritis model mice [18]	8-week-old mice with OP and/or OA	Bone micro-architecture by micro-computed tomography (μCT), tartrate-resistant acid-phosphatase 5b (TRAP5b) levels, von Frey test, Runx2, Osterix, Osteocalcin, Rankl levels	- Higher TRAP5b level in OP and OP/OA mice - Lower N values in OP mice - Higher expression levels of Runx2, Osterix Rankl and osteocalcin in OP mice - Higher pain-like behaviors in OA/OP mice - Cartilage degeneration in OA/OP mice	Treatment with ALN alleviated pain-like behaviors in OA/OP mice, with effects lasting for at least 4 weeks, and inhibited the increase in osteoclast numbers in these mice
miRNA-92a-3p regulates osteoblast differentiation in patients with concomitant limb fractures and traumatic brain injury (TBI) via integrin-binding sialoprotein (IBSP)/PI3K-AKT inhibition [19]	30 patients (10 healed fractures, 10 isolated fractures, 10 concomitant fractures and TBI) and C57BL/6J mice	RT-PCR for miRNA-92a-3p levels, X-rays, micro-CT scans, ALP and alizarin red staining and Western blotting	- Higher miRNA-92a-3p levels in patients with concomitant fractures and TBI. - Faster fracture healing in the TBI and fracture group - Increased osteoblast differentiation and matrix mineralization in vitro with miRNA-92a-3p overexpression - Enhanced bone callus formation in mice models - miRNA-92a-3p directly targets and inhibits IBSP, leading to increased PI3K-AKT signaling	Exploiting miRNA-92a-3p to modulate IBSP and PI3K-AKT pathways for enhanced osteoblast differentiation and accelerated fracture healing
A magnesium metal–organic Frameworks (Mg-MOFs) based multifunctional medicine for the treatment of osteoporotic pain [20]	Mg-MOF-74 loaded with ketoprofen	Drug release and ion release experiments Cell experiments in vitro	- Significant reduction in cyclooxygenase 2 (COX2) expression (pain-related gene) - Increased osteogenic cytokines - Decreased inflammatory factors	Ketoprofen alleviates pain and inflammation by inhibiting COX2, and Mg promotes bone formation and reduces inflammation. Provides combined pain relief and bone growth stimulation, effectively addressing OP through a multifunctional drug delivery system
Characterization of neuropathic component of back pain in patients with osteoporotic vertebral fractures [21]	72 patients with chronic back pain and OVFs	Changes in pain severity (BPI), neuropathic pain assessment (PD-Q, LANSS), vertebral fracture characteristics (DXA), and ADL impact	- Mild pain (BPI severity: 4.0 ± 1.8) and low interference with ADL (BPI interference: 4.3 ± 2.1) - 23.6% of patients showed probable neuropathic pain (LANSS), 5.5% showed neuropathic component (PD-Q) - Thoracic OVFs were associated with higher prevalence of neuropathic pain compared to lumbar fractures	Anti-osteoporotic treatments (bisphosphonates, teriparatide, denosumab) may help manage pain by reducing inflammation and improving bone healing
A multicenter, randomized, double-blind, placebo-controlled clinical study of Jianyao Migu granules (JYMGGs) in the treatment of osteopenic low back pain [22]	108 patients with primary osteopenic low back pain	Changes in BMD, visual analog scale (VAS) score, Oswestry disability index (ODI), and bone turnover markers	- Lumbar vertebral BMD increased by 2.70% (JYMGGs) vs. 0.93% (placebo) - Femoral neck BMD increased by 1.96% (JYMGG) vs. a decrease of 0.09% (placebo) - Increased ALP, osteocalcin, Procollagen 1 Intact N-Terminal Propeptide (P1NP), and serum C-terminal telopeptide of type I collagen (β-CTX) levels in both groups - Thyroid-stimulating hormone (TSH) levels significantly higher in JYMGG group - Serum calcium and 25-OH-vitamin D (VITD) increased	JYMGG improved VAS and ODI scores.
Wingless-related integration site (Wnt) signaling pathway inhibitors, sclerostin and Dickkopf-1 (DKK-1), correlate with pain and bone pathology in patients with Gaucher disease (GD) [23]	20 healthy controls and GD patients aged 18 to 68 years, grouped by bone mineral density (BMD)	Sclerostin, DKK-1, and Receptor activator of nuclear factor kappa-Β ligand (RANKL) levels in blood; BMD measurements	- Elevated sclerostin is linked to reduced BMD, bone pain, bone marrow infiltration in patients with GD. - Altered sclerostin/DKK-1 ratio correlates with reduced BMD. - DKK-1 did not show significant correlation with pain or bone pathology in GD patients.	Sclerostin and DKK-1 inhibit the Wnt/β-catenin signaling pathway. High sclerostin levels serve as a marker for bone pathology and pain in GD, with potential for use in assessing disease severity and progression.
The Effect of group training or spinal orthosis on quality of life and potential plasma markers of pain in older women with osteoporosis [24]	113 women, median age 76 years with self-reported OP and back pain with or without vertebral fracture	Downton Fall Risk Index (DFRI), quality of life questionnaire of the European Foundation for OP (QUALEFFO-41), The Short Form health survey (SF-36), calcitonin gene-related peptide (CGRP), substance P (SP), interleukin-6 (IL-6), Isometric back extensor strength measured by Digi-Max	- Highest fall risk in spinal orthosis group; lowest in exercise group - Spinal orthosis group showed better mobility in QUALEFFO-41 compared to Controls - Worsened activities of daily living in Exercise group compared to Controls - IL-6 significantly lower in Spinal Orthosis group compared to Controls and Exercise group - Exercise group had worsened SF-36 Role Emotional domain - Spinal Orthosis group had improved QUALEFFO-41 Pain domain at 3 months	IL-6 reduction noted in spinal orthosis group. Evaluated impacts of spinal orthosis and group training on QoL and plasma markers; limited QoL improvements noted.
Effect of high-intensity laser therapy versus shockwave therapy on selected outcome measures in osteoporotic long-term hemiparetic patients: a randomized control trial [25]	140 hemiplegic patients with OP, aged 60–70	VAS, OSI Short Form of Berg Balance Scale (SFBBS), QUALEFFO-41 evaluation	- Post-treatment, significant differences in VAS, overall stability index, SFBBS, and QUALEFFO-41 between groups - high-intensity laser (HIL) and shockwave (SW) groups showed better VAS scores than the control group - Significant improvement in overall stability index, SFBBS, and QUALEFFO-41 for HIL and SW groups compared to control	Combining High-Intensity Laser Therapy (HILT) and Extracorporeal Shockwave Therapy (ESWT) with traditional physical therapy is more effective than physical therapy alone. This approach is associated with improved outcomes, potentially through mechanisms involving endothelial nitric oxide synthase (eNOS) and vascular endothelial growth factor (VEGF).
Sclerostin and Wnt Signaling in Idiopathic Juvenile Osteoporosis (JIO) [26]	13 patients affected by IJO	Quantification of sclerostin in osteocytes and evaluation of biochemical parameters including calcium (Ca), phosphorous (P), parathyroid hormone (PTH)	- Sclerostin expression was increased in 8 of 13 patients (46%) - Bone histomorphometry showed diminished trabecular bone formation and mineralization - Sclerostin staining correlated with serum alkaline phosphatase and negatively correlated with bone volume - Elevated sclerostin was associated with low Wnt signaling, as indicated by high levels of phosphorylated β-catenin and low levels of unphosphorylated β-catenin - Sclerostin appears to inhibit Wnt signaling, contributing to diminished bone formation in IJO patients	Wnt signaling pathway involvement with sclerostin as an inhibitor
Therapeutic effects of mechanical stress-induced C2C12-derived exosomes on glucocorticoid-induced osteoporosis through miR-92a-3p/Phosphatase and tensin homolog (PTEN)/serine/threonine protein kinase (AKT) signaling pathway [27]	Human bone marrow mesenchymal stem cells (hBMSCs) and mouse myoblasts (C2C12)	Western blotting, real-time polymerase chain reaction (RT-PCR), alkaline phosphatase (ALP) activity assay, alizarin red staining, microCT, HE staining, Masson staining, and immunohistochemistry	- In vitro: exosomes under mechanical stress enhance osteogenesis in BMSCs - In vivo: These exosomes treat glucocorticoid-induced OP (GIOP) in mice	Using mechanical stress-induced exosomes (exosome–MS) to improve bone formation and treat glucocorticoid-induced OP by delivering miR-92a-3p. This approach stimulates bone repair and regeneration by affecting the miR-92a-3p/PTEN/AKT signaling pathway.

Abbreviations: hBMSCs: human bone marrow mesenchymal stem cells; C2C12: mouse myoblast cell line; RT-PCR: real-time polymerase chain reaction; ALP: alkaline phosphatase; GIOP: glucocorticoid-induced osteoporosis; exosome–MS: exosomes under mechanical stress; COX2: cyclooxygenase-2; DKK-1: Dickkopf-1; GD: Gaucher Disease; BMD: bone mineral density; RANKL: receptor activator of nuclear factor kappa-B ligand; GWAS: genome-wide association studies; SNPs: single-nucleotide polymorphisms; EPDR1: Mammalian Ependymin-Related Protein 1; PKDCC: Protein Kinase Domain-Containing Cytoplasm; SPTBN1: Spectrin Beta Non-Erythrocytic 1; JYMGG: Jianyao Migu granules; VAS: visual analog scale; ODI: Oswestry disability index; TSH: thyroid-stimulating hormone; VITD: Vitamin D; DXA: dual-energy X-ray absorptiometry; P1NP: Procollagen Type 1 N-terminal Propeptide; OST: osteocalcin; BSAP: bone-specific alkaline phosphatase; β-CTx: beta-C-terminal telopeptide; AEs: adverse events; TBI: traumatic brain injury; IBSP: integrin-binding sialoprotein; SDF-1: Stromal Cell-Derived Factor 1; PMOP: postmenopausal osteoporosis; TNF-α: tumor necrosis factor alpha; QUALEFFO-41: Quality of Life Questionnaire of the European Foundation for Osteoporosis; CGRP: calcitonin gene-related peptide; SP: substance P; IL-6: Interleukin 6; SFBBS: short form of Berg balance scale; HIL: high-intensity laser; HILT: high-intensity laser therapy; ESWT: extracorporeal shockwave therapy; eNOS: endothelial nitric oxide synthase; Plekha2: Pleckstrin Homology Domain-Containing A2; Plekhb1: Pleckstrin Homology Domain-Containing B1; Pnpla7: Patatin-Like Phospholipase Domain-Containing 7; Scd: Stearoyl-CoA Desaturase; Mgst3: Microsomal Glutathione S-Transferase 3; Tsnax: Translin-Associated Factor X; Prkcz: Protein Kinase C Zeta; Gna11: G Protein Subunit Alpha 11; Col4a1: Collagen Type IV Alpha 1 Chain; Sox6: SRY-Box Transcription Factor 6; Ace: Angiotensin I-Converting Enzyme; Syk: Spleen-Associated Tyrosine Kinase; Tgfb3: Transforming Growth Factor Beta 3; cGMP-PKG: Cyclic GMP-Dependent Protein Kinase; Rap1: Ras-Related Protein 1; AMPK: AMP-Activated Protein Kinase; SCD: Stearoyl-CoA Desaturase; MGST3: Microsomal Glutathione S-Transferase 3; TSNAX: Translin-Associated Factor X; TGFB3: Transforming Growth Factor Beta 3; SOX6: SRY-Box Transcription Factor 6; GNA11: G Protein Subunit Alpha 11; cGMP-PKG: Cyclic GMP-Dependent Protein Kinase; AMPK: AMP-Activated Protein Kinase; OVCF: osteoporotic vertebral compression fractures; ZOL: zoledronic acid; OA: osteoarthritis; μCT: micro-computed tomography; TRAP5b: tartrate-resistant acid phosphatase 5b; Runx2: Runt-related transcription factor 2; ALN: alendronate; BPI: brief pain inventory; PD-Q: painDETECT questionnaire; LANSS: Leeds assessment of neuropathic symptoms and signs pain scale; ADL: activities of daily living.
